# Real-time PCR has advantages over culture-based methods in identifying major airway bacterial pathogens in chronic obstructive pulmonary disease: Results from three clinical studies in Europe and North America

**DOI:** 10.3389/fmicb.2022.1098133

**Published:** 2023-02-24

**Authors:** Sonia Schoonbroodt, Jean-Laurent Ichanté, Sophie Boffé, Nathalie Devos, Jeanne-Marie Devaster, Laura Taddei, Simona Rondini, Ashwani Kumar Arora, Thierry Pascal, Ludovic Malvaux

**Affiliations:** ^1^GSK, Rixensart, Belgium; ^2^GSK, Wavre, Belgium; ^3^GSK, Siena, Italy

**Keywords:** bacterial identification, culture, PCR, sensitivity, *Haemophilus influenzae*, *Moraxella catarrhalis*, *Streptococcus pneumoniae*, chronic obstructive pulmonary disease

## Abstract

**Introduction:**

We compared the performance of real-time PCR with culture-based methods for identifying bacteria in sputum samples from patients with chronic obstructive pulmonary disease (COPD) in three studies.

**Methods:**

This was an exploratory analysis of sputum samples collected during an observational study of 127 patients (AERIS; NCT01360398), phase 2 study of 145 patients (NTHI-004; NCT02075541), and phase 2b study of 606 patients (NTHI-MCAT-002; NCT03281876). Bacteria were identified by culture-based microbiological methods in local laboratories using fresh samples or by real-time PCR in a central laboratory using frozen samples. *Haemophilus influenzae* positivity with culture was differentiated from *H. haemolyticus* positivity by microarray analysis or PCR. The feasibility of bacterial detection by culture-based methods on previously frozen samples was also examined in the NTHI-004 study.

**Results:**

Bacterial detection results from both culture-based and PCR assays were available from 2,293 samples from AERIS, 974 from the NTHI-004 study, and 1736 from the NTHI-MCAT-002 study. Quantitative real-time PCR (qPCR) showed higher positivity rates than culture for *H. influenzae* (percentages for each study: 43.4% versus 26.2%, 47.1% versus 23.6%, 32.7% versus 10.4%) and *Moraxella catarrhalis* (12.9% versus 6.3%, 19.0% versus 6.0%, 15.5% versus 4.1%). In the NTHI-004 and NTHI-MCAT-002 studies, positivity rates were higher with qPCR for *Streptococcus pneumoniae* (15.6% versus 6.1%, 15.5% versus 3.8%); in AERIS, a lower rate with qPCR than with culture (11.0% versus 17.4%) was explained by misidentification of *S. pseudopneumoniae/mitis* isolates *via* conventional microbiological methods. Concordance analysis showed lowest overall agreement for *H. influenzae* (82.0%, 75.6%, 77.6%), due mainly to culture-negative/qPCR-positive samples, indicating lower sensitivity of the culture-based methods. The lowest positive agreement (culture-positive/qPCR-positive samples) was observed for *S. pneumoniae* (35.1%, 71.2%, 71.2%). Bacterial load values for each species showed a proportion of culture-negative samples with a load detected by qPCR; for some samples, the loads were in line with those observed in culture-positive samples. In the NTHI-004 study, of fresh samples that tested culture-positive, less than 50% remained culture-positive when tested from freeze/thawed samples. In the NTHI-004 study, of fresh samples that tested culture-positive, less than 50% remained culture-positive when tested from freeze/thawed samples.

**Discussion:**

Real-time PCR on frozen sputum samples has enhanced sensitivity and specificity over culture-based methods, supporting its use for the identification of common respiratory bacterial species in patients with COPD.

## Introduction

Chronic obstructive pulmonary disease (COPD) is a common condition characterized by persistent respiratory symptoms, often punctuated by acute exacerbations that can lead to hospitalization and a faster decline in lung function (Criner et al., 2015; Global Initiative for Chronic Obstructive Lung Disease, 2021). Different studies, including the observational Acute Exacerbation and Respiratory InfectionS in COPD (AERIS) study, have shown an association between exacerbation state and increased prevalence of airway bacteria, most commonly non-typeable *Haemophilus influenzae* (NTHi) and *Moraxella catarrhalis*, with some reports of an association with *Streptococcus pneumoniae* also (Garcha et al., 2012; Molyneaux et al., 2013; Huang et al., 2014; Millares et al., 2014; Wang et al., 2015; Wilkinson et al., 2017; Jubinville et al., 2018; Mayhew et al., 2018; Weeks et al., 2021). In the AERIS study, changes in the yearly COPD exacerbation rate were associated with changes in *H. influenzae* colonization (Wilkinson et al., 2019a), and nearly all (99%) *H. influenzae* isolates were non-typeable (Wilkinson et al., 2017).

A multi-component investigational vaccine was developed to reduce the frequency of acute exacerbations of COPD (AECOPD) associated with NTHi (Leroux-Roels et al., 2016), followed by a second related vaccine (NTHi-Mcat vaccine) to prevent NTHi-and *M. catarrhalis*-associated exacerbations (Van Damme et al., 2019). Both vaccines contain three conserved surface NTHi proteins, while the NTHi-Mcat vaccine also contains a surface protein from *M. catarrhalis* (Leroux-Roels et al., 2016; Van Damme et al., 2019). A phase 2 study of the NTHi vaccine (NTHI-004) and phase 2b study of the NTHi-Mcat vaccine (NTHI-MCAT-002) showed immunogenicity and no safety concerns when either vaccine was administered to patients with COPD (Wilkinson et al., 2019b; Andreas et al., 2022). In the NTHI-MCAT-002 study, while vaccination did not reduce the frequency of moderate/severe exacerbations, with no difference between groups in rate of AECOPD associated with NTHi or *M. catarrhalis*, observations suggested possible reductions in severe exacerbations and related hospitalizations in the vaccinated group versus placebo (Andreas et al., 2022).

In the AERIS, NTHI-004, and NTHI-MCAT-002 studies, sputum samples were collected from patients at regular intervals and at exacerbation (Wilkinson et al., 2017, 2019b; Andreas et al., 2022). Bacteria in freshly collected sputum were identified in local laboratories using conventional culture-based microbiological methods that were not harmonized. In parallel, frozen dithiothreitol (DTT)-treated sputum samples were transported to a central laboratory and analyzed by real-time PCR assay. Various studies have shown that molecular techniques, such as PCR, have better specificity and sensitivity than conventional culture-dependent methods in the detection of airway bacteria in patients with COPD (Eser et al., 2012; Garcha et al., 2012; Bafadhel et al., 2015; Gadsby et al., 2015; Wilkinson et al., 2017). Also, culture-based methods require fresh samples and their reliability can be affected by antibiotic treatment (Oliver, 2010; Wu et al., 2014) as well as the microbiologist’s skills in phenotypic identification methods. In contrast, a PCR assay can be used on frozen samples in a central location where it has been well characterized, thus avoiding the risk of variations in microbiological methods among individual local laboratories.

We now report on the performance of the microbiological assays used in the detection of bacteria in sputum samples in the AERIS, NTHI-004, and NTHI-MCAT-002 studies (Wilkinson et al., 2017, 2019b; Andreas et al., 2022). Quantitative and qualitative *H. influenzae, M. catarrhalis*, and *S. pneumoniae* identification results obtained with real-time PCR are compared with those obtained with culture-based methods. Additionally, using data from the NTHI-004 study, we examine the feasibility of freezing sputum samples before bacterial detection by culture in a central laboratory. Qualitative *Pseudomonas aeruginosa, Staphylococcus aureus*, and *Streptococcus pyogenes* identification data by PCR assay are also assessed from the three studies.

**Figure 1:**
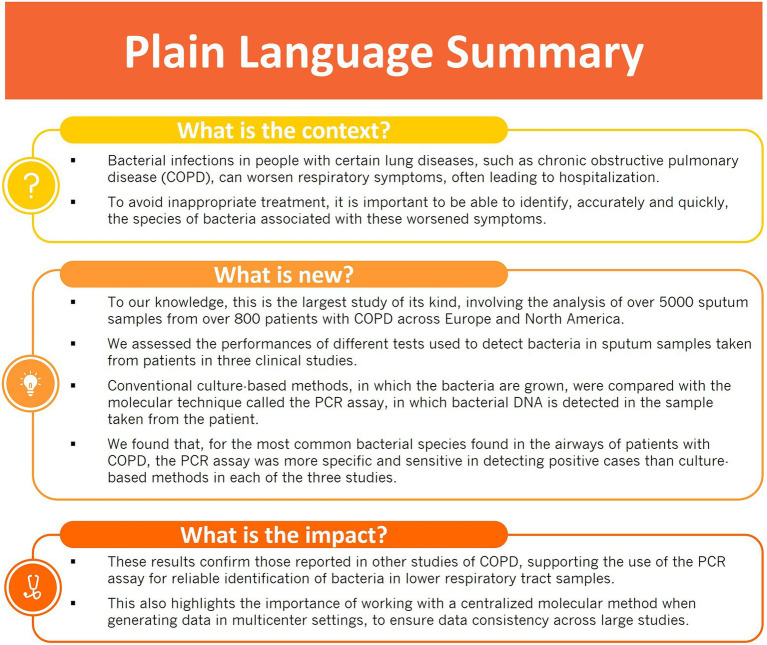
Plain language summary.

Figure 1 provides a plain language summary of the findings from these assessments.

## Materials and methods

### Sputum sample collection and processing

The sputum samples were collected in three clinical studies of adults with COPD: the prospective observational cohort study, AERIS, of 127 patients (NCT01360398; Wilkinson et al., 2017), the phase 2 placebo-controlled NTHI-004 study of an investigational NTHi vaccine in 145 patients (NCT02075541; Wilkinson et al., 2019b), and the phase 2b placebo-controlled NTHI-MCAT-002 study of an investigational NTHi-Mcat vaccine in 606 patients (NCT03281876; Andreas et al., 2022). The AERIS study was conducted in the United Kingdom between June 2011 and June 2014, the NTHI-004 study in the UK and Sweden between July 2014 and April 2017, and the NTHI-MCAT-002 study in Belgium, Canada, France, Germany, Italy, Spain, United Kingdom, and United States between November 2017 and March 2020. Study summaries are available^1^ (study identifiers 114378, 200157, and 207489) and methods and results on primary and secondary endpoints of the studies have been published (Wilkinson et al., 2017; Mayhew et al., 2018; Osman et al., 2018; Wilkinson et al., 2019b; Malvisi et al., 2021; Andreas et al., 2022).

Sputum samples were obtained by spontaneous expectoration or induced, as per the investigator’s judgement, at regular intervals during each study and at each exacerbation visit, and were processed according to standard methods, as described previously (Wilkinson et al., 2017, 2019b; Andreas et al., 2022). An acute exacerbation was defined as worsening of at least two major symptoms (dyspnea, sputum volume, and sputum purulence) or worsening of at least one major symptom and one minor symptom (sore throat, cold symptoms, fever, increased cough, and increased wheeze) for at least two consecutive days.

### Culture-based bacteriological methods and confirmatory assays

**Figure 2:**
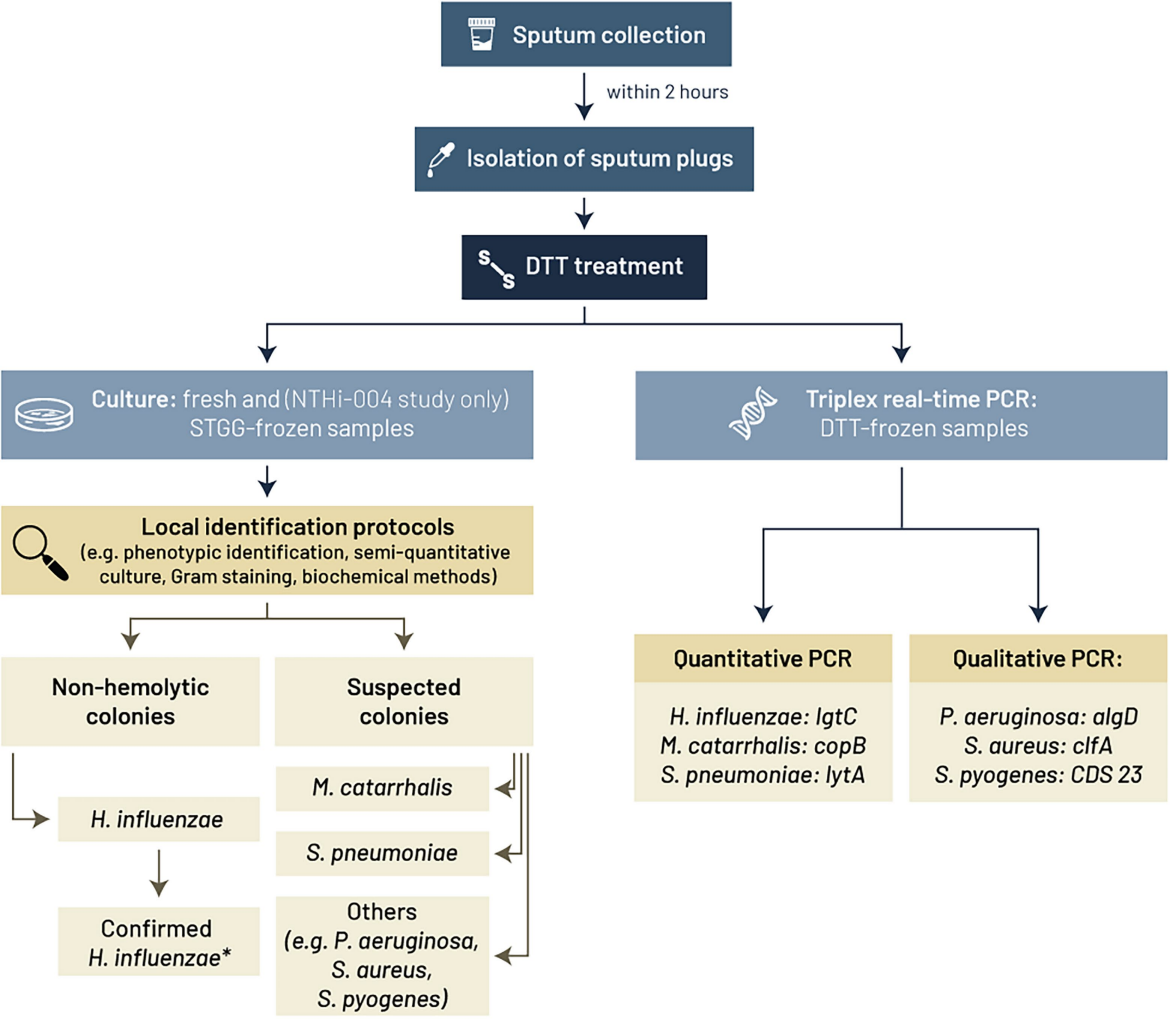
Culture-based and PCR methods used in the three clinical studies (AERIS, NTHI-004, and NTHI-MCAT-002) to identify *Haemophilus influenzae*, *Moraxella catarrhalis*, *Streptococcus pneumoniae*, *Pseudomonas aeruginosa*, *Staphylococcus aureus*, and *Streptococcus pyogenes*. * Samples confirmed as *H. influenzae* positive after differentiation from other *Haemophilus* species (e.g., *H. haemolyticus*) by (NTHI-004 study) Senti-HI microarray analysis on stored *H. influenzae* sweep or (AERIS and NTHI-MCAT-002 studies) *lgtC*/*P6* qualitative real-time PCR assay. *algD,* GDP mannose dehydrogenase encoding gene; *CDS* 23, coding sequence 23; *clfA,* clumping factor A encoding gene; *copB*, copB outer membrane protein encoding gene; DTT, dithiothreitol; h, hours; *lgtC*, lipo-oligosaccharide glycosyltransferase encoding gene; *lytA*, autolysin encoding gene; STGG, skim milk-tryptone-glucose-glycerol medium.

Freshly collected sputum samples were treated with DTT at 49 local microbiological laboratories associated with the clinical sites (one site for AERIS study, 15 for NTHI-004 study, and 67 for NTHI-MCAT-002 study). Bacterial species were identified by standard culture-based identification procedures (Murray et al., 2007), as summarized in Figure 2, and according to each local laboratory’s routine methods, with minor adaptations (relating to, for example, the agar plate used for culture, sputum DTT pre-treatment, and semi-quantitative culture data reporting). Identification protocols included phenotypic characterization, semi-quantitative culture, and other methods such as Gram staining and biochemical methods. In the AERIS and NTHI-MCAT-002 studies, qualitative real-time PCR was used to further discriminate bacterial isolates of *H. influenzae* from *H. haemolyticus* by assessing the presence of two genes: *P6* (outer membrane protein gene), which is conserved among *H. influenzae* and *H. haemolyticus*, and *lgtC* (lipo-oligosaccharide glycosyltransferase gene), which is ubiquitous in all *H. influenzae* but only 2% prevalent in *H. haemolyticus* (McCrea et al., 2008; Sandstedt et al., 2008; Abdeldaim et al., 2009). In the NTHI-004 study, culture plate sweeps were taken from presumptive *H. influenzae*-positive sputum in order to confirm *H. influenzae* positivity. This was part of an extended investigation of sputum sweep samples by Senti-HI molecular serotyping microarray analysis (data not shown) at BUGS Bioscience laboratory (London, United Kingdom). Culture plate sweeps from presumptive *H. influenzae*-positive sputum were stored in skim milk-tryptone-glucose-glycerol (STGG) medium (O'Brien et al., 2001; Kaijalainen et al., 2004) and genomic DNA extracted. Confirmation of species was based on the detection of species-specific gene targets for *H. influenzae,* in parallel with no detection of species-specific gene targets for eight other relevant *Haemophilus* species.

In the NTHI-004 study, a fraction of all DTT-treated sputum samples was stored in STGG medium before freezing and was shipped to the central laboratory for bacterial identification using standard culture-based methods.

Bacterial identification data generated using culture-based methods were qualitative (positive/negative). The bacterial load of *H. influenzae, M. catarrhalis,* and *S. pneumoniae* in cultured samples was estimated using a semi-quantitative quadrant assessment method (Murdoch et al., 2017) adapted according to local laboratory practices, using a ‘0, Few, 1+, 2+, 3+’ scale, with ‘0’ indicating absence and ‘3+’ corresponding to the highest load (bacterial presence in at least three of four quadrants of the agar plate). The ‘Few’ category, which applied when only few, sparsely spread colonies were observed in any quadrant of the plate, was not recorded in the NTHI-004 study, according to the routine practices of the local laboratories.

### PCR identification methods

Frozen aliquots of DTT-treated sputum samples were transported to the testing laboratory and analyzed by real-time PCR assay (Figure 2). The methods used for nucleic acid extraction and PCR are summarized in Table 1 and primers and probes sequences are provided in Supplementary Table 1.

**Table 1:** Methods used for the triplex real-time PCR assays.

A. Triplex quantitative real-time PCR (qPCR) assay to identify *Haemophilus influenzae*, *Moraxella catarrhalis*, and *Streptococcus pneumoniae*			
Study	AERIS	NTHI-004	NTHI-MCAT-002
Nucleic acid extraction kit and protocol	MagNA Pure 96 equipment (Roche)	Kingfisher equipment (Life Technologies)	MagNA Pure 96 equipment (Roche)
	MagNA Pure 96 DNA and Viral NA small volume kit (viral NA universal protocol)	LSI MagVet Universal Isolation kit	MagNA Pure 96 DNA and Viral NA large volume kit (pathogen universal 500 protocol)
Real-time qPCR reagent	Taqman Fast Advanced Multiplex Universal Master Mix (Life Technologies)		
*H. influenzae* target gene	*lgtC*		
*M. catarrhalis* target gene	*copB*		
*S. pneumoniae* target gene	*lytA*		
Sputum-DTT input volume	200 μl	100 μl	500 μl
Nucleic acid eluate volume	50 μl	80 μl	100 μl
Nucleic acid volume input in qPCR reaction	2 μl	2 μl	2 μl
Copies/qPCR to copies/ml conversion factor	Copies/ml = Copies/qPCR * 125	Copies/ml = Copies/qPCR * 400	Copies/ml = Copies/qPCR * 667
Positivity cut-off (assay LOD)			
*H. influenzae*	2,000 copies/ml	16,400 copies/ml	1,561 copies/ml
*M. catarrhalis*	15,000 copies/ml	44,800 copies/ml	927 copies/ml
*S. pneumoniae*	12,875 copies/ml	12,400 copies/ml	1,161 copies/ml
Testing laboratory	DDL Diagnostic	GSK Vaccines	GSK Vaccines
B. Triplex qualitative real-time PCR assay to identify *Pseudomonas aeruginosa*, *Staphylococcus aureus*, and *Streptococcus pyogenes*			
Study	AERIS	NTHI-004	NTHI-MCAT-002
Nucleic acid extraction kit and protocol	MagNA Pure 96 equipment (Roche)	Kingfisher equipment (Life Technologies)	MagNA Pure 96 equipment (Roche)
	MagNA Pure 96 DNA and Viral NA small volume kit (viral NA universal protocol)	LSI MagVet Universal Isolation Kit	MagNA Pure 96 DNA and Viral NA large volume kit (pathogen universal 500 protocol)
Real-time PCR reagent	Taqman Fast Advanced Multiplex Universal Master Mix (Life Technologies)		
*P. aeruginosa* target gene	*algD*		
*S. aureus* target gene	*clfA*		
*S. pyogenes* target gene	*CDS 23*		
Sputum-DTT input volume	200 μl	100 μl	500 μl
Nucleic acid eluate volume	50 μl	80 μl	100 μl
Nucleic acid volume input in PCR reaction	2 μl	2 μl	2 μl
Positivity cut-off (assay LOD)*			
*P. aeruginosa*	16,625 copies/ml	21,600 copies/ml	768 copies/ml
*S. aureus*	17,500 copies/ml	20,000 copies/ml	1,108 copies/ml
Testing laboratory	DDL Diagnostic	GSK Vaccines	GSK Vaccines

copB, copB outer membrane protein encoding gene; DTT, dithiothreitol; lgtC, lipo-oligosaccharide glycosyltransferase encoding gene; LOD, limit of detection; lytA, autolysin encoding gene. algD, GDP mannose dehydrogenase encoding gene; CDS 23, coding sequence 23; clfA, clumping factor A encoding gene; DTT, dithiothreitol; LOD, limit of detection. *S. pyogenes not detected in sputum samples.

Two triplex real-time PCR assays were employed that were developed by GSK and characterized in the AERIS study by DDL Diagnostic Laboratory (Rijswijk, the Netherlands) and in the NTHI-004 and NTHI-MCAT-002 studies by the central GSK laboratory (Wavre, Belgium). Following total nucleic acid extraction (see Table 1), a quantitative real-time PCR (qPCR) assay (Andreas et al., 2022) amplified DNA fragments of *H. influenzae, M. catarrhalis,* and *S. pneumoniae* using the TaqMan Fast Advanced Master Mix kit (Life Technologies) on Viia7 or QuantStudio 7 equipment (Life Technologies). A second PCR assay was a qualitative assay that amplified *P. aeruginosa, S. aureus,* and *S. pyogenes* DNA using the same nucleic acids and the same PCR reagents and equipment (Life Technologies). The protocol for the second assay was essentially the same as for the first, with adaptation of the primers and probes sequences (Supplementary Table 1).

For the qPCR assay, three sets of primers and probes were designed from the conserved region of *lgtC* for *H. influenzae* (McCrea et al., 2008), the outer membrane protein *copB* gene for *M. catarrhalis* (Greiner et al., 2003), and the autolysin A gene (*lytA*) for *S. pneumoniae*. The *lytA* primers and probe sequences corresponded to those used in the lytA-CDC assay (Carvalho et al., 2007). The presence of *S. pyogenes, S. aureus*, and *P. aeruginosa* was determined using a qualitative real-time triplex PCR assay targeting conserved regions of the coding sequence 23 gene (*CDS23*), the clumping factor A encoding gene (*clfA*), and the GDP mannose dehydrogenase encoding gene (*algD*), respectively. *S. pyogenes* was not detected in any of the sputum samples.

PCR identification results were quantitative (copies/ml) or qualitative (positive/negative). Only qualitative data are available for *P. aeruginosa* and *S. aureus*. The sample was considered positive when the measured load was equal to or above the assay cut-off corresponding to the limit of detection (LOD) of the assay. LODs, expressed in copies/ml of DTT-treated sputum, were defined during characterization of the technical performance of the qPCR assay. Over time, there was re-assessment of some assay parameters, including LOD and quantitation limits, due to improvement of the methods (e.g., updated methods for nucleic acids extraction). There were consequent differences in LOD values between studies (see Table 1). The concentration of *H. influenzae*, *M. catarrhalis*, and *S. pneumoniae* DNA (bacterial load, copies/ml) in each sample was inferred from the calibration curve made from serial dilutions of a plasmid containing the sequences targeted by the assay and converted from copies/qPCR to copies/ml of DTT-treated sputum samples.

The specificity of these assays was verified theoretically (i.e., bioinformatic analyses) and experimentally (on related and unrelated bacteria and viruses commonly found in respiratory samples). No significant signal above the assays positivity cut-offs was observed and the sequencing of the PCR products generated from sputum samples confirmed that amplified material corresponded accurately to the reference sequences of the targeted pathogens.

### Statistical analyses

This technical comparison was an exploratory analysis of data from sputum samples taken during the AERIS study (Wilkinson et al., 2017), NTHI-004 study (Wilkinson et al., 2019b), and NTHI-MCAT-002 study (Andreas et al., 2022), regardless of sputum quality and per-protocol defined cohorts. Positivity rates and their 95% confidence intervals (CIs) were calculated for the results of each assay. Concordance and agreement analyses were conducted on the qualitative identification results from samples for which both culture-based and PCR assay results were available. Overall agreement, positive agreement, and negative agreement were calculated as described in the footnote to Table 2. Dissymmetry in the number of discordant samples was evaluated by McNemar’s test. In the analysis of bacterial load, samples with qPCR values above 0 copies/ml were considered for median computation and were plotted as a function of semi-quantitative culture results.

**Table 2:** Concordance analysis between bacterial pathogen identification results obtained by sputum culture and PCR assay (samples with both culture and PCR results).

		Number of samples				Overall agreement^a^ (%)	Positive agreement^b^ (%)	Negative agreement^c^ (%)	*P* value^d^
Pathogen	Study	Culture-negative, PCR-negative	Culture-negative, PCR-positive	Culture-positive, PCR-negative	Culture-positive, PCR-positive				
*Haemophilus influenzae* ^e^	AERIS	1,173	367	9	538	81.98	98.35	76.17	<0.0001
	NTHI-004	501	229	5	223	75.57	97.81	68.63	<0.0001
	NTHI-MCAT-002	1,140	379	1	176	77.59	99.44	75.05	<0.0001
*Moraxella catarrhalis*	AERIS	1,824	143	4	128	93.00	96.97	92.73	<0.0001
	NTHI-004	773	127	2	55	86.52	96.49	85.89	<0.0001
	NTHI-MCAT-002	1,462	201	4	68	88.18	94.44	87.91	<0.0001
*Streptococcus pneumoniae*	AERIS	1,631	103	237	128	83.80	35.07	94.06	<0.0001
	NTHI-004	790	108	17	42	86.94	71.19	87.97	<0.0001
	NTHI-MCAT-002	1,445	221	19	47	86.14	71.21	86.73	<0.0001
*Pseudomonas aeruginosa*	AERIS	1,967	35	10	113	97.88	91.87	98.25	0.0002
	NTHI-004	918	13	4	21	98.22	84.00	98.60	0.0490
	NTHI-MCAT-002	1,593	49	8	85	96.71	91.40	97.02	<0.0001
*Staphylococcus aureus*	AERIS	1,993	23	33	76	97.36	69.72	98.86	0.2288
	NTHI-004	874	46	10	26	94.14	72.22	95.00	<0.0001
	NTHI-MCAT-002	1,517	108	26	85	92.28	76.58	93.35	<0.0001

^a^ Calculated as [(Culture-positive, PCR-positive) + (Culture-negative, PCR-negative)] divided by [(Culture-positive, PCR-positive) + (Culture-negative, PCR-negative) + (Culture-positive, PCR-negative) + (Culture-negative, PCR-positive)]. ^b^ Calculated as (Culture-positive, PCR-positive) divided by [(Culture-positive, PCR-positive) + (Culture-positive, PCR-negative)]. ^c^ Calculated as (Culture-negative, PCR-negative) divided by [(Culture-negative, PCR-negative) + (Culture-negative, PCR-positive)]. ^d^ Difference between culture-negative/PCR-positive samples and culture-positive/PCR-negative samples: McNemar’s test. ^e^ Samples confirmed as positive after differentiation from H. haemolyticus by Senti-HI microarray analysis or lgtC/P6 real-time PCR assay.

## Results

### Sampling

The total number of sputum samples from which bacterial detection results were available from both culture-based and PCR assays were 2,293 sputum samples from 127 patients in the AERIS study (years 1 and 2), 974 sputum samples from the NTHI-004 study (488 from 73 patients who received the investigational NTHi vaccine and 486 from 72 patients in the placebo group), and 1736 sputum samples from the NTHI-MCAT-002 study (878 from 304 patients who received the NTHi-Mcat vaccine and 858 from 302 patients who received placebo).

### Bacterial identification

**Figure 3:**
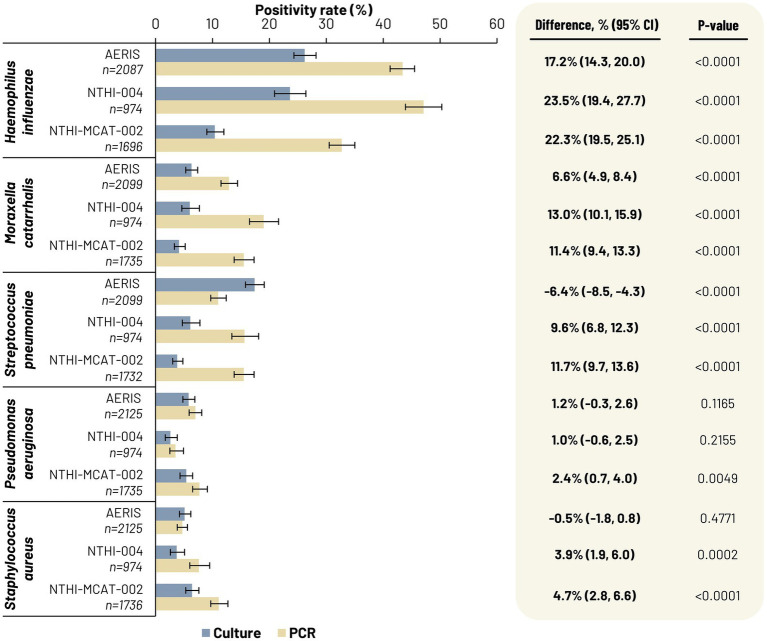
Percentage of culture-positive sputum samples derived from freshly collected samples assessed in local laboratories (grey bars) and percentage of PCR-positive sputum samples derived from frozen samples and assessed centrally (light brown bars). 95% CI, 95% confidence interval; n, number of samples for which there were both valid culture and PCR results (POS/NEG) for each targeted pathogen.

For *H. influenzae* and *M. catarrhalis* in all three studies, and *S. pneumoniae* in the NTHI-004 and NTHI-MCAT-002 studies, concordance analysis showed overall agreement of culture and qPCR data was between 75 and 93% (Table 2). Overall agreements were mainly impacted by the low negative agreement. Indeed, more samples were positive when identification was performed using qPCR, with statistically significant (*p* < 0.0001) differences between positivity rates for samples assessed by culture versus those assessed by qPCR (Figure 3). In the concordance analysis, culture-negative/qPCR-positive results were associated with significant *p*-values (p < 0.0001; McNemar’s test), indicating increased sensitivity with qPCR compared to culture-based methods for the three bacterial species (Table 2).

The concordance analysis also showed, for *H. influenzae*, *M. catarrhalis*, and *S. pneumoniae*, few culture-positive and qPCR-negative samples, apart from for *S. pneumoniae* in the AERIS study (Table 2). Moreover, the positivity rate for *S. pneumoniae* in the AERIS study was significantly higher (*p* < 0.0001) with culture identification than with qPCR (Figure 3). This discrepancy between culture and qPCR assay results observed with *S. pneumoniae* in the AERIS study was investigated further *via* various molecular techniques and mass spectrometry (see Supplementary material). This confirmed that the high rate of false-positive samples by culture-based methods was due to misidentification of samples containing *Streptococcus pseudopneumoniae* or *Streptococcus mitis* species.

For *P. aeruginosa,* the positivity rate was significantly higher (*p* = 0.0049) with PCR than with culture in the NTHI-MCAT-002 study only, while for *S. aureus*, a statistically significant difference was only observed in the NTHI-004 study (*p* = 0.0002) and the NTHI-MCAT-002 study (*p* < 0.0001), with higher positivity rates with PCR (Figure 3). Concordance analysis showed overall agreement of culture and PCR data was between 92% and 98% (Table 2). The difference between culture-negative/PCR-positive samples and culture-positive/PCR-negative samples was statistically significant (*p* < 0.05; McNemar’s test) in all three studies, apart from the discordant results for *S. aureus* in the AERIS study (Table 2).

The NTHI-004 study also evaluated the feasibility of freezing sputum samples before bacterial detection by culture in a central laboratory. The comparison of two culture-based methods for identifying *H. influenzae*, *M. catarrhalis*, and *S. pneumoniae* showed lower positivity rates for STGG-frozen samples processed centrally than for fresh samples tested locally (Supplementary Table 2). Of fresh samples that tested *H. influenzae*-positive by culture, thawed frozen samples remained positive for 39.5% of samples and positive agreement was 47.1% for *M. catarrhalis* and 18.5% for *S. pneumoniae* (Supplementary Table 3).

### Bacterial load

**Figure 4:**
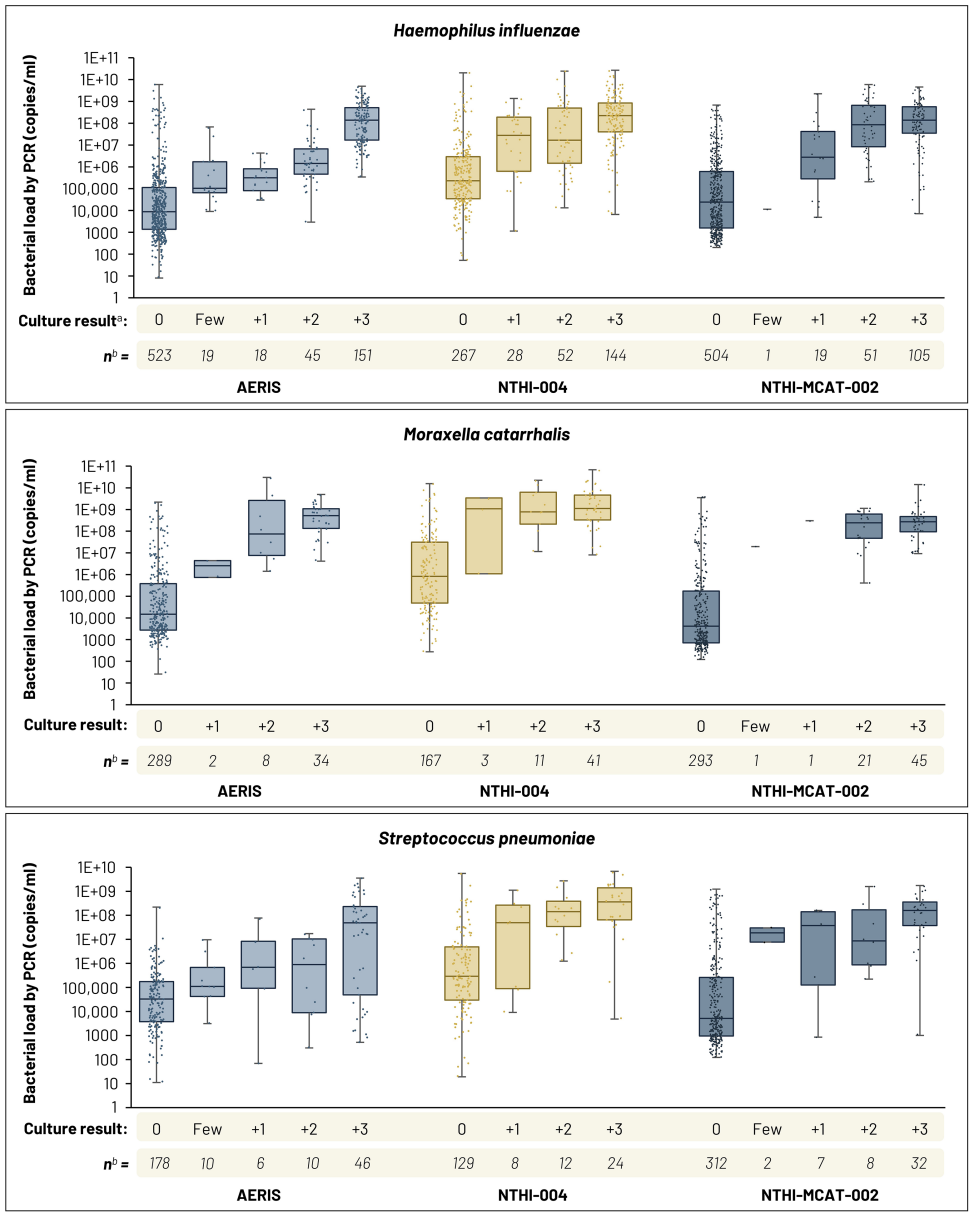
Box and dot plots of bacterial load results by quantitative real-time PCR (qPCR) as a function of semi-quantitative culture results. Median, first and third quartiles, and minimum and maximum data shown. ^a^ Culture-positive samples confirmed by Senti-HI microarray or *lgtC/P6* real-time PCR assay. ^b^ Samples with qPCR loads above >0 copy/reaction and associated with a semi-quantitative culture result. See Table 1 for qPCR positivity cut-offs for *H. influenzae*, *M. catarrhalis,* and *S. pneumoniae*. Bacterial load in cultured samples estimated using the quadrant assessment method, according to a ‘0, Few, 1+, 2+, 3+’ scale, with ‘0’ indicating absence and ‘3+’ corresponding to the highest load. ‘Few’ category not recorded in NTHI-004 study.

Bacterial load data generated by qPCR were plotted against the semi-quantitative results from culture-based methods for *H. influenzae*, *M. catarrhalis*, and *S. pneumoniae*. Figure 4 shows data for samples with qPCR loads above >0 copy/reaction and associated with a semi-quantitative culture result. There was a positive trend between the qPCR load and semi-quantitative culture results. For each species, the median load calculated by qPCR in sputum samples was higher in culture-positive samples than in culture-negative samples (Figure 4). Most culture-negative samples were associated with null bacterial loads by qPCR (not shown in Figure 4). For the AERIS study, of 1,540, 1,967, and 1,734 samples that were culture-negative for *H. influenzae*, *M. catarrhalis*, and *S. pneumoniae*, respectively, 1,017 (66.0%), 1,678 (85.3%), and 1,556 (89.7%) had null bacterial loads by qPCR. For the NTHI-004 study, of 730, 900, and 898 samples that were culture-negative for *H. influenzae*, *M. catarrhalis*, and *S. pneumoniae*, respectively, 463 (63.4%), 733 (81.4%), and 769 (85.6%) had null bacterial loads by qPCR. Similarly, for the NTHI-MCAT-002 study, of 1,519, 1,663, and 1,666 culture-negative samples for *H. influenzae*, *M. catarrhalis*, and *S. pneumoniae*, respectively, 1,015 (66.8%), 1,370 (82.4%), and 1,354 (81.3%) had null bacterial loads by qPCR. Therefore, across all three studies, 33–37% (*H. influenzae*), 15–19% (*M. catarrhalis*), and 10–19% (*S. pneumoniae*) of culture-negative samples were detected by qPCR assay (below or above the positivity cut-off), with loads for some samples in line with those observed in culture-positive samples (Figure 4).

## Discussion

We compared the performance of culture-based and PCR assays in identifying bacterial airway pathogens in sputum samples taken in three clinical studies of patients with COPD. In each study, two triplex real-time PCR assays were used, developed by GSK: a qPCR assay to detect and quantify *H. influenzae*, *M. catarrhalis,* and *S. pneumoniae* from sputum total nucleic acids, involving hydrolysis probes to maximize specificity, and a qualitative PCR assay to detect *P. aeruginosa*, *S. aureus*, and *S. pyogenes*. *S. pyogenes* was not identified in sputum samples from any of the studies. The three studies were conducted over the period 2011 to 2020, during which time the PCR assays were improved, such as in extraction methods for nucleic acids, leading to adjustments in LOD.

Our results show that real-time qPCR has higher specificity and sensitivity than culture-based methods for the detection of the most frequent bacterial species identified: *H. influenzae*, *M. catarrhalis*, and *S. pneumoniae*. The bacterial load analyses for these three species showed that some samples negative by culture contained a significant amount (over 10 million copies/ml) of bacterial DNA. Moreover, for *P. aeruginosa* and *S. aureus*, analysis of over 1,700 samples in the NTHI-MCAT-002 study showed significantly higher positivity rates with PCR than with culture-based detection methods for both species, although in the other two studies the difference was only significant for *S. aureus* in the NTHI-004 study. Overall, however, these data confirm previous reports of higher sensitivity with the PCR assay than with culture-based methods not only in COPD but also other conditions, such as otitis media and cystic fibrosis (Eser et al., 2012; Garcha et al., 2012; Bafadhel et al., 2015; Gadsby et al., 2015; Wilkinson et al., 2017; Gavillet et al., 2022). For example, one study of the detection of *P. aeruginosa* and *S. aureus* in cystic fibrosis found significantly lower detection of both lung pathogens by culture, which often did not detect either pathogen despite being found repeatedly by qPCR (Gavillet et al., 2022). In our study, qPCR positivity rates were around two-to four-fold higher for *H. influenzae* and *M. catarrhalis* in all three studies and for *S. pneumoniae* in the NTHI-004 and NTHI-MCAT-002 studies. In the concordance analysis, the lowest percentage positive agreement was for *S. pneumoniae* in the AERIS study (35%), reflecting the proportion of qPCR-negative samples among the culture-positive ones. Further analysis of this discrepant result led to the re-identification of a significant number of isolates, initially attributed to be *S. pneumoniae* isolates, as *S. pseudopneumoniae* or *S. mitis* by molecular techniques. The reason for this observation in the AERIS study may be related to characteristics of locally circulating strains or the specificity of the culture methods used to identify *S. pneumoniae* in the AERIS study laboratory; positive agreement in the other two studies was 71%. This suggests the culture-based method may have, in some settings, low specificity for *S. pneumoniae*.

A lack of specificity with culture-based methods is also demonstrated by the need to discriminate *H. influenzae* from *H. haemolyticus* isolates in presumptive *H. influenzae*-positive samples by using molecular methods (Murphy et al., 2007), as confirmed by qPCR or microarray in the three studies analyzed. Of the bacterial species assessed, the concordance analysis showed the lowest percentage overall agreement (76–82%) for *H. influenzae* (confirmed samples), due mainly to samples that were negative with culture-based detection but positive with qPCR. However, the presence of culture-negative samples associated with high bacterial DNA loads suggests this may have been partly due to the presence of non-culturable but viable bacteria (Oliver, 2010) or poor sensitivity of the culture method used. For example, use of bacitracin agar increases identification rates for *H. influenzae* (Harris et al., 2017) but this was not used in all laboratories. There is also a possibility that, although patients were instructed not to take an antibiotic before site visits, antibiotic treatment before sputum collection could have had an impact on the bacterial culture results (Brown and Spiegel, 2019). In this case, bacteria may not have been capable of growing on an agar plate but would have been detected by real-time PCR.

Our data suggest important advantages related to the specificity and sensitivity of real-time PCR over conventional culture-based methods for the assessment of airway bacteria in patients with COPD. Molecular assays can be performed on frozen sputum samples (Zhao et al., 2011; Cuthbertson et al., 2014), whereas culture needs to be done shortly after sputum collection to ensure sample integrity and bacteria viability (Baron et al., 2013). Frozen samples can be processed in a central laboratory with a well characterized PCR-based method, while fresh samples processed *via* conventional microbiological methods may require multiple local laboratories to avoid loss of viability. These local laboratories may not use exactly the same methods (see Figure 2), affecting the consistency of results (Hogardt et al., 2009). Culture of freeze/thawed samples in a central laboratory was examined in the NTHI-004 study as an option to avoid unharmonized methods across local laboratories. This analysis of the impact of freezing sputum samples before species identification by culture-based methods used STGG as storage medium since preserved *H. influenzae*, *M. catarrhalis,* and *S. pneumoniae* isolate viability had been reported with its use (Kaijalainen et al., 2004). However, the culture-positivity rate in STGG-frozen sputum samples was only around 50% or less of that in fresh samples, showing this is not a feasible option for evaluating sputum samples, thus providing further support for PCR testing of frozen samples in a central laboratory. The ability to freeze sputum samples is a particular advantage in phase 3 clinical trials of COPD patients, which generally require large numbers of sites to enroll a sufficient number of patients (Tashkin et al., 2008; Wedzicha et al., 2008; Calverley et al., 2009; Albert et al., 2011; Wedzicha et al., 2013; Pascoe et al., 2016). However, central analysis of frozen samples is associated with additional shipment costs and there is a risk of inappropriate sample management during the transport or freezing procedure, although there is also a management risk associated with fresh samples, for example, if not processed within the appropriate time window. Additionally, antibiotic usage before sputum sample collection can have an impact on the reliability of bacterial data obtained by culture (Wu et al., 2014; Mammen and Sethi, 2016; Jacobs et al., 2018), and the identification of pathogens by culture can be complicated by species overgrowth and contamination by commensal bacteria, while PCR allows direct species detection irrespective of these circumstances (Scoleri et al., 2016). PCR also has many advantages in terms of easy evaluation of bacterial load, it is relatively inexpensive, and it can be used to process more samples simultaneously than culture. These advantages assume the PCR assay has been appropriately designed (with highly specific oligonucleotide selection) and well characterized in terms of assay parameters, including LOD and quantitation limits, as was done for the PCR assays used in the AERIS, NTHI-004, and NTHI-MCAT-002 studies.

A major strength of these analyses is the large number (totaling 5,003) and multinational origin (Europe and North America) of sputum samples assessed for bacterial detection results from both culture-based and PCR assays. Also, the triplex real-time PCR assays used in each study were essentially the same, with any differences related to improvements made over time. The results of these analyses are limited by the possibility of false-positives resulting from the detection of low amounts of DNA, associated with dead bacteria, by qPCR. However, this was mitigated in these studies by using a LOD for each qPCR target as positivity cut-off (i.e., a sample was considered positive for a pathogen by qPCR if the observed load is equal to or above the corresponding LOD). We found many culture-and PCR-negative samples had PCR signals detected below the positivity cut-off, suggesting that using a PCR positivity cut-off is meaningful to limit the proportion of false-positives. Although qPCR can detect both viable and non-viable bacteria (Rudi et al., 2005), we found the bacterial loads measured by qPCR generally mirrored those measured by culture. Nevertheless, it would have been of interest to determine if samples found negative with culture-based techniques but positive on qPCR assay contained viable bacteria that were not culturable, as reported in other studies (Stenfors and Räisänen, 1992; Oliver, 2010; Lee and Bae, 2018), as well as the rate of non-viable bacteria present in samples recorded as qPCR-positive.

In conclusion, these results encourage the use of real-time PCR assays for the identification of respiratory bacteria in patients with COPD. PCR assay addresses some of the limitations of conventional culture-based methods in terms of specificity and sensitivity in detecting bacterial infection. PCR has additional advantages, including the ability to be performed in a centralized location on frozen samples and the capacity to detect viable but non-culturable bacteria. This supports the use of well characterized molecular methods for the identification and quantification of bacteria in future studies of patients with COPD, especially when working in a multicenter setting requiring sample testing in multiple laboratories.

## Data availability statement

The datasets presented in this article are not readily available because anonymized individual participant data and study documents can be requested for further research from www.clinicalstudydatarequest.com.

## Ethics statement

The studies involving human participants were reviewed and approved by the South West Hampshire Research Ethics Committee (Research Ethics Committee reference number: 11/H0502/9) for the AERIS study; the Southampton and South West Hampshire Research Ethics Committee, United Kingdom, and Regional Research Ethics Committee, Gothenburg, Sweden for the NTHI-004 study; the ethics committee of each participating center for the NTHI-MCAT-002 study. The patients/participants provided their written informed consent to participate in this study.

## Author contributions

SS, ND, J-MD, LT, SR, AKA, TP, and LM were involved in analysis conception and design. SB, ND, J-MD, LT, SR, AKA, TP, and LM were involved in acquisition and generation of data. All authors performed the data analysis and/or data interpretation. ND and LM provided materials for the analyses. All authors contributed to the article and approved the submitted version.

## Funding

GlaxoSmithKline Biologicals SA funded the AERIS, NTHI-004, and NTHI-MCAT-002 clinical studies and was involved in all stages of study conduct, including analysis of the data. GlaxoSmithKline Biologicals SA also took in charge all costs associated with the development and publication of this manuscript.

## Conflict of interest

SS, J-LI, SB, ND, J-MD, LT, SR, AKA, TP, and LM are employed by GSK. SS, ND, J-MD, LT, TP, and LM hold shares in GSK. J-MD is also a designated inventor on patents owned by GSK.

## Publisher’s note

All claims expressed in this article are solely those of the authors and do not necessarily represent those of their affiliated organizations, or those of the publisher, the editors and the reviewers. Any product that may be evaluated in this article, or claim that may be made by its manufacturer, is not guaranteed or endorsed by the publisher.

## Footnotes

^1^www.gsk-studyregister.com

## Abbreviations

AECOPD: acute exacerbations of chronic obstructive pulmonary disease
AERIS: Acute Exacerbation and Respiratory InfectionS in COPD
*algD*: GDP mannose dehydrogenase encoding gene
*CDS 23*: coding sequence 23 gene
*clfA*: clumping factor A encoding gene
*copB*: copB outer membrane protein encoding gene
COPD: chronic obstructive pulmonary disease
DTT: dithiothreitol
*lgtC*: lipo-oligosaccharide glycosyltransferase encoding gene
LOD: limit of detection
*lytA*: autolysin encoding gene
Mcat: *Moraxella catarrhalis*
NTHi: non-typeable *Haemophilus influenzae*
PCR: Polymerase Chain Reaction
qPCR: quantitative real-time PCR
STGG: skim milk-tryptone-glucose-glycerol
