# Supplementary material for: Real-time PCR has advantages over culture-based methods in identifying major airway bacterial pathogens in chronic obstructive pulmonary disease: Results from three clinical studies in Europe and North America
Source: Front Microbiol. 2023 Feb 24;13:1098133. doi: 10.3389/fmicb.2022.1098133 (PMC10000296; doi:10.3389/fmicb.2022.1098133)
Supplement: Supplementary file 1 [file Data_Sheet_1.docx]

Supplementary Material

# Additional *Streptococcus* Species Identification Tests in the AERIS Study

Presence of *Streptococcus pneumoniae* in sputum samples by culture was first indicated by phenotypic observation. Then, α-hemolytic colonies were selected and underwent an exact measure of optochin susceptibility, with *S. pneumoniae* presence shown by a zone of inhibition of ≥14 mm around a 5 µg optochin disk (*DIATABS* Optochin, ROSCO, Denmark) in a 5% CO_2_ environment. If optochin inhibition was intermediary (<14mm), a bile solubility assay was performed and bile soluble isolates were considered as *S. pneumoniae*. On re-testing of all samples identified as containing *S. pneumoniae*, a proportion of the culture-positive sputum samples was negative for *lytA*, indicating an absence of *S. pneumoniae* and hence discordance with the culture result. An additional investigational analysis was conducted on discordant samples for *S. pneumoniae*, as summarized in Supplementary Figure 1. This analysis was performed on isolates from a subset of randomly selected sputum samples from year 1 of the study by monoplex *lytA* quantitative real-time PCR (qPCR), 16S rRNA gene restriction fragment length polymorphism (RFLP), matrix-assisted laser desorption ionization-time of flight (MALDI-TOF) mass spectrometry, and whole genome sequencing.

For the monoplex *lytA* qPCR, *S. pneumoniae* isolates were grown overnight at 37°C on solid Columbia agar with 5% horse blood (Biomérieux, Marcy l’Etoile, France). Nucleic acids were extracted as described above. qPCR was performed using *lytA*-specific primers and probe (Carvalho et al., 2007) (sequences identical to those in triplex qPCR assay), with the *TaqMan* Fast Advanced Master Mix Kit (Life Technologies) on a *ViiA7* instrument (Applied Biosystems, CA, USA). The *lytA* qPCR primers and probe matched a region where polymorphisms exist between *S. pneumoniae* and other closely related streptococci.

For 37 *lytA* qPCR-negative isolates (Supplementary Figure 1), optochin susceptibility was re-tested as follows. The isolates (inoculum density, 0.5 McFarland) were grown overnight at 35–36°C in an atmosphere containing 5% CO_2_ on Columbia agar containing 5% sheep blood and one optochin disk 10 µg (*DIATABS* Optochin, ROSCO, Denmark). Pneumococci showed an inhibition zone ≥18 mm around the optochin disc, while streptococci showed an inhibition zone <16 mm. The test was repeated if the inhibition zone was 16–17 mm.

The 16S rRNA gene RFLP was performed on isolates according to the method described by Scholz et al. (2012): the gene was partially amplified with primers ATGAGTTGCGAACGGGTGAGTAA and ATTGCCGAAGATTCCCTACTGCT and PCR amplicons (286 base pairs [bp]) were restricted using the BsiHKAI enzyme. RFLP signature 164 bp plus 122 bp is characteristic of *S. pneumoniae*; 286 bp is characteristic of *S. pseudopneumoniae* or *S. mitis*.

Species identification of isolates was performed using MALDI-TOF mass spectrometry (*MALDI Biotyper*, Bruker Daltonics GmbH, Bremen, Germany), according to the manufacturer’s instructions. Single colonies were transferred onto a target plate and overlaid with matrix solution. *FlexControl* software (version 3.4) was used for the measurements and the spectra were analyzed using *BioTyper* software (version 3.1.66) and database MBT DB-5627. Interpretation of the results obtained was limited by the similarity in MALDI-TOF spectra for *S. pneumoniae* and *S. pseudopneumoniae*. In addition, the database in place contained 30 *S. pneumoniae* isolates and only one *S. pseudopneumoniae* isolate. Therefore, the ability to discriminate between *S. pneumoniae* and *S. pseudopneumoniae* was poor.

For whole genome sequencing, genomic DNA from the isolates was purified further with *AMPure* XP Beads (Beckman Coulter, CA, USA) before being processed for library generation using the *Nextera* XT (Illumina, CA, USA), following manufacturer recommendations and using 0.25 ng of genomic DNA and 15 PCR cycles. Sequencing was performed on an Illumina *MiSeq* instrument using the *MiSeq* Reagent v3 600-cycle kit according to the manufacturer’s recommendations for paired end sequencing (2 × 300 cycles). Multilocus sequence typing (MLST) and identification of genes for speciation was performed using SRST2 (version 0.1.5) (Inouye et al., 2014) on whole genome sequencing streptococcal isolates and the *MiSeq* Illumina platform, with a target of 30 x coverage of MLST genes (*S. pneumoniae* scheme). Here, paired-end FASTQs were mapped to reference sequences. All parameters were used by default except for minimum coverage (minimum percentage coverage cut-off for gene reporting was, by default, 90%). To ensure the presence of genes (complete, partial, or recombinant sequences), a minimum coverage cut-off of 60% was used. Differentiation between *S. pneumoniae* and *S. pseudopneumoniae* was based on *lytA* presence or absence, the presence of a *S. pneumoniae*-specific cytosine at position 203 in the 16S rRNA gene (with adenosine presence indicating another streptococcal species) (Scholz et al., 2012), and presence of nucleotides specific for *S. pneumoniae* in the 313-bp *recA* fragment, as indicated by GTTCCT nucleotide sequence at positions 97, 160, 199, 247, 250, and 280 (Zbinden et al., 2011). Non-*S. pneumoniae* isolates were mapped using Burrows-Wheeler Aligner (Li and Durbin, 2010) against a public bacterial sequences database (National Center for Biotechnology Information nucleotide database: https://www.ncbi.nlm.nih.gov/genome/microbes/).

Whole genome sequencing revealed that 316/373 (84.7%) isolates from qPCR-positive sputum samples displayed *lytA* and 16s rRNA gene sequences characteristic of *S. pneumoniae*, while the sequences of the remaining isolates were characteristic of *S. pseudopneumoniae/mitis*. One isolate per negative sputum sample was also sequenced. Among these 58 isolates, all but one displayed *lytA* and 16s rRNA gene sequences characteristic of *S. pseudopneumoniae/mitis.*

All isolates from 21 selected qPCR-positive or -negative sputum samples were re-analyzed with *lytA* qPCR and 16s rRNA gene RFLP. All displayed the expected signal, except for one positive isolate from a negative sputum sample. Thirty-seven *lytA* qPCR-negative isolates from five qPCR-negative sputum samples (from three patients) were analyzed by MALDI-TOF mass spectrometry and optochin susceptibility. All were identified as *S. pneumoniae* by MALDI-TOF mass spectrometry but the database was not optimized for *S. pseudopneumoniae*/*mitis* discrimination. Analysis of optochin susceptibility showed mixed results, with isolates from individual patients displaying susceptibility, resistance, or intermediate susceptibility (Supplementary Figure 1). Whole genome sequencing showed isolates from the same three patients displayed no *lytA* gene sequences characteristic of *S. pneumoniae* but the sequences were characteristic of *S. pseudopneumoniae/mitis.*

**References**

Carvalho, M. G. S., Tondella, M. L., McCaustland, K., Weidlich, L., McGee, L., Mayer, L. W., Steigerwalt, A., Whaley, M., Facklam, R. R., Fields, B., Carlone, G., Ades, E. W., Dagan, R., and Sampson, J. S. (2007). Evaluation and improvement of real-time PCR assays targeting *lytA*, *ply*, and *psaA* genes for detection of pneumococcal DNA. *J. Clin. Microbiol.* 45, 2460-2466.

Inouye, M., Dashnow, H., Raven, L. A., Schultz, M. B., Pope, B. J., Tomita, T., Zobel, J., and Holt, K. E. (2014). SRST2: Rapid genomic surveillance for public health and hospital microbiology labs. *Genome Med.* 6, 90.

Li, H., Durbin, R.. (2010). Fast and accurate long-read alignment with Burrows-Wheeler transform. *Bioinformatics* 26, 589-595.

Scholz, C. F., Poulsen, K., and Kilian, M. (2012). Novel molecular method for identification of *Streptococcus pneumoniae* applicable to clinical microbiology and 16S rRNA sequence-based microbiome studies. *J. Clin. Microbiol*. 50, 1968-1973.

Zbinden, A., Köhler, N., and Bloemberg, G. V. (2011). *recA*-based PCR assay for accurate differentiation of *Streptococcus pneumoniae* from other viridans streptococci. *J. Clin. Microbiol.* 49, 523-527.

# SUPPLEMENTARY FIGURE 1 Procedure for additional *Streptococcus* species investigations and results (AERIS study full cohort, year 1). This analysis was conducted on a subset of 97 samples originating from 23 patients and corresponding to 858 isolates to further investigate the observation of a higher positivity rate for *S. pneumoniae* by culture compared to PCR in the AERIS study.


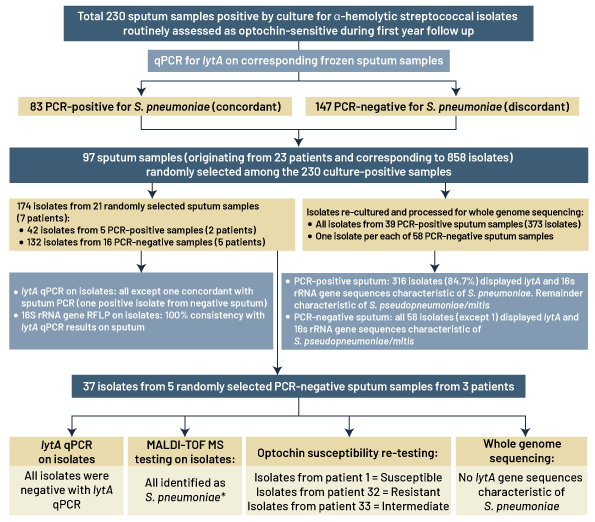


*lytA*, autolysin encoding gene; MALDI-TOF MS, matrix-assisted laser desorption ionization-time of flight mass spectrometry; qPCR, quantitative real-time PCR; RFLP, restriction fragment length polymorphism.

*The ability to discriminate between *S. pneumoniae* and *S. pseudopneumoniae* is poor with MALDI-TOF MS.

SUPPLEMENTARY TABLE 1 Primer and probe sequences for the quantitative real-time PCR assay used to detect *H. influenzae, M. catarrhalis,* and *S. pneumoniae*, and qualitative real-time PCR assay used to detect *P. aeruginosa, S. aureus,* and *S. pyogenes*.

| Targeted pathogen/Trial | Targeted gene | Name of primers | Sequence | Amplimer size |
| --- | --- | --- | --- | --- |
| *Haemophilus influenzae* | *lgtC* | Forward Primer | 5’-TNGCNACTTACGCTAGACTAAATTTAACTAA-3’ | 150 bp |
|  |  | Reverse Primer | 5’-TATCTCGACATICTGCCAAATAATA-3’ |  |
|  |  | Probe | 5’-/56-FAM/C**CA**AAGTTCTTGAAG**T**G**A**A/3BHQ_1/-3’ |  |
| *Moraxella catarrhalis/*AERIS | *copB* | Forward Primer | 5’-ATTCGTGGCATGGGTCATA-3’ | 125 bp |
|  |  | Reverse Primer | 5’-TGTACCGAAACNACTTTGACCAT-3’ |  |
|  |  | Probe | 5’-HEX/CACCAAGGTCGCTTTATGCTAGACCC/IAbRQSp-3’ |  |
| *M. catarrhalis*/* NTHI-004 &  NTHI-MCAT-002 | *copB* | Forward Primer | 5’-GTGAGTGCCGCTTTTACAACC-3’ | 72 bp |
|  |  | Reverse Primer | 5’-TGTATCGCCTGCCAAGACAA-3’ |  |
|  |  | Probe | 5’-/5HEX/TGCTTTTGC/ZEN/AGCTGTTAGCCAGCCTAA/3IABkFQ/-3’ |  |
| *Streptococcus pneumoniae* | *lytA* | Forward Primer | 5’-ACGCAATCTAGCAGATGAAGCA-3’ | 73 bp |
|  |  | Reverse Primer | 5’-TCGTGCGTTTTAATTCCAGCT-3’ |  |
|  |  | Probe | 5’-/5Cy5/TGCCGAAAACGCTTGATACAGGGAG/3IAbRQSp/-3’ |  |
| *Streptococcus pyogenes* | *CDS23* | Forward Primer | 5’-GTAGGGATTGAACTTGATGATCTCT-3’ | 100bp |
|  |  | Reverse Primer | 5'-GCCTTACCGCATCTTCTTGA-3' |  |
|  |  | Probe | 5’-HEX/CCATTATCTCAGCAATACTGGCTGACAGA/IAbRQSp-3’ |  |
| *Staphylococcus aureus* | *clfA* | Forward Primer | 5’-CAGTATCATCTGTAAATTCACCTCAAA-3’ | 111 bp |
|  |  | Reverse Primer | 5’-TCTGTGGAGCTGATTCATTGTT-3’ |  |
|  |  | Probe | 5’-FAM/CTTGCGTTGTTGAAACATTTTCCGCAT/BHQ1-3’ |  |
| *Pseudomonas aeruginosa* | *algD* | Forward Primer | 5’-GCATCCAAGGTCGGGTTA-3’ | 87 bp |
|  |  | Reverse Primer | 5’-GGCACTCTAATNGGGCAAGTTA-3’ |  |
|  |  | Probe | 5’-/5Cy5/TGAAACCCACCTGAAACATTAAAGGGC/IAbRQSp-3’ |  |

N underlined corresponds to any of the following nucleotides A,C,T,G; Locked Nucleic Acid (LNA) is indicated in bold and underlined; bp, base pair.

**M. catarrhalis* PCR was optimized for NTHI-004 and NTHI-MCAT-002 studies to limit the risk of false positive results due to putative aspecific signals raised following to updated bioinformatic analyses.

# SUPPLEMENTARY TABLE 2 Percentage of culture-positive sputum samples derived from freshly collected samples assessed in local laboratories or STGG-frozen sputum samples assessed in the central laboratory, and percentage of quantitative real-time PCR-positive sputum samples derived from frozen samples and assessed centrally in NTHI-004 study (974 samples).

| Pathogen | Positivity rate, % (95% CI) | | |
| --- | --- | --- | --- |
|  | Culture  (fresh samples) | Culture  (STGG-frozen samples) | PCR  (frozen samples) |
| *Haemophilus influenzae* | 23.6 (20.9, 26.4)^a^ | 10.5 (8.5, 12.7)^a^ | 47.1 (43.9, 50.3) |
| *Moraxella catarrhalis* | 6.0 (4.6, 7.7) | 3.3 (2.2, 4.7) | 19.0 (16.5, 21.6) |
| *Streptococcus pneumoniae* | 6.1 (4.7, 7.8) | 1.4 (0.7, 2.4) | 15.6 (13.4, 18.1) |

^a^ Samples confirmed positive by Senti-HI microarray.

95% CI, 95% confidence interval. STGG, skim milk-tryptone-glucose-glycerol medium.

# SUPPLEMENTARY TABLE 3 Concordance analysis in NTHI-004 study of bacterial pathogen identification results obtained by culture-based assay in local laboratories (using fresh sputum samples) or in the central laboratory (using STGG-frozen sputum samples).

|  | Number of sputum samples with culture-based assay result | | | | |  |  |  |
| --- | --- | --- | --- | --- | --- | --- | --- | --- |
| Pathogen | Total | Culture/fresh-negative, Culture/frozen-negative | Culture/fresh-negative, Culture/frozen-positive | Culture/fresh-positive, Culture/frozen-negative | Culture/fresh-positive, Culture/frozen-positive | Overall agreement^a^ (%) | Positive agreement^b^ (%) | Negative agreement^c^ (%) |
| *Haemophilus influenzae*^d^ | 858 | 641 | 7 | 127 | 83 | 84.38 | 39.52 | 98.92 |
| *Moraxella catarrhalis* | 857 | 802 | 4 | 27 | 24 | 96.38 | 47.06 | 99.50 |
| *Streptococcus pneumoniae* | 857 | 801 | 2 | 44 | 10 | 94.63 | 18.52 | 99.75 |

^a^ Calculated as ((Culture/fresh-positive, culture/frozen-positive) + (Culture/fresh-negative, culture/frozen-negative)) divided by ((Culture/fresh-positive, culture/frozen-positive) + (Culture/fresh-negative, culture/frozen-negative) + (Culture/fresh-positive, culture/frozen-negative) + (Culture/fresh-negative, culture/frozen-positive)).

^b^ Calculated as (Culture/fresh-positive, culture/frozen-positive) divided by ((Culture/fresh-positive, culture/frozen-positive) + (Culture/fresh-positive, culture/frozen-negative)).

^c^ Calculated as (Culture/fresh-negative, culture/frozen-negative) divided by ((Culture/fresh-negative, culture/frozen-negative) + (Culture/fresh-negative, culture/frozen-positive)).

^d^ Samples confirmed positive by Senti-HI microarray.

STGG, skim milk-tryptone-glucose-glycerol medium.
